# Rheumatoid nodule presenting as Morton’s neuroma

**DOI:** 10.1007/s10195-012-0215-x

**Published:** 2012-11-08

**Authors:** S. Chaganti, S. Joshy, K. Hariharan, M. Rashid

**Affiliations:** 1 Department of Orthopaedics, Royal Gwent Hospital, Newport, UK; 2 Derriford Hospital, 20 Echo Crescent, Plymouth, PL6 3UQ UK; 3 Department of Histopathology, Royal Gwent Hospital, Newport, UK

**Keywords:** Morton’s neuroma, Intermetatarsal neuroma, Rheumatoid nodule, Rheumatoid synovitis, Forefoot pain

## Abstract

Among 101 feet that presented with symptoms and signs similar to Morton’s neuroma, intermetatarsal rheumatoid nodules were found in five feet (three patients). Two patients had bilateral involvement. Histology of the excised tissue showed the presence of a rheumatoid nodule and Morton’s neuroma in four feet and a rheumatoid nodule with unremarkable nerve bundles in one. A rheumatoid nodule can coexist with Morton’s neuroma, as seen in our patients, and the presentation is often similar to that of a Morton’s neuroma. Our patients were rendered asymptomatic with surgical treatment and went on to have appropriate management of rheumatoid arthritis. Rheumatoid nodule should be considered in the differential diagnosis of Morton’s neuroma in not only rheumatoid arthritis patients but also asymptomatic patients who have never been tested for rheumatoid antibodies.

## Introduction

Morton’s neuroma is a common paroxysmal neuralgia affecting the forefoot, typically in the third interdigital space. The clinical syndrome of Morton’s neuroma was described over a century ago, but its etiopathology remains poorly understood [1, 8, 9]. Recent studies suggest that Morton’s neuroma is a mechanically induced degenerative neuropathy which has a strong predilection for the third common digital nerve [10].

Clinical examination is the most sensitive and specific, and is superior to imaging modalities like ultrasound or magnetic resonance imaging scan [7]. Various treatment options have been described in the literature for the treatment of Morton’s neuroma, including infiltration of a local anaesthetic and steroid combination into the interdigital space, sonography-guided alcohol injection, and surgery. The results of local steroid injections [2] and ultrasound-guided alcohol injections [4] are comparable to those of surgery, but limited literature is available on long-term results. Some authors still recommend therapeutic injections prior to surgery [2]. However, surgical resection has shown good long-term results, with improvement in 80 % of cases [6].

Rheumatoid synovitis and nodules producing symptoms mimicking Morton’s neuroma have been reported in the literature [1, 3, 9], but are still rare. Most of the cases reported in the literature were found in rheumatoid arthritis patients [1, 7, 9]. We report three patients (five feet) presenting with symptoms and signs of Morton’s neuroma due to underlying rheumatoid nodules. Two patients had bilateral involvement. Histology of the excised tissue showed the presence of a rheumatoid nodule and Morton’s neuroma in four feet and a rheumatoid nodule and unremarkable nerve bundles in one. Out of these three patients, one had a previous history of rheumatoid arthritis. Our patients were rendered asymptomatic with surgical treatment and went on to have appropriate management of rheumatoid arthritis.

## Case report

Informed consent was obtained from the patients prior to being included in the case series.

**Fig. 1:**
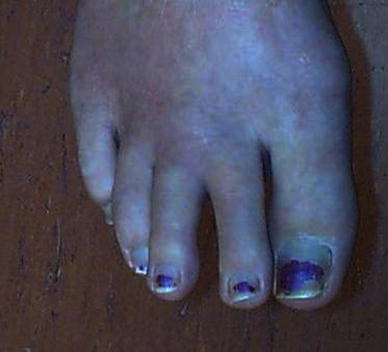
Fullness in the second intermetatarsal space, with splaying of the surrounding toes

**Case 1.** A 38-year-old lady presented to the foot and ankle clinic with pain and a burning sensation over the third intermetatarsal space of the right foot and the second intermetatarsal space of the left foot. She was healthy with no past medical history of note. Clinical examination revealed fullness in the interdigital space with splaying of the corresponding toes (Fig. 1) and tenderness over the plantar aspect of the corresponding intermetatarsal space with a positive Mulder’s click. A clinical diagnosis of Morton’s neuroma was made. The patient underwent surgery through a single dorsal linear incision in the corresponding intermetarsal space. Intraoperatively, a large soft tissue mass 2 cm in size involving the digital nerve and capsule of the second metatarsophalangeal (MTP) joint was noted on the left side, along with necrosis of the underlying fat pad. A soft tissue swelling originating from the dorsum of the capsule of the third MTP joint, filling the intermetatarsal space, was noted on the right side.

**Fig. 2:**
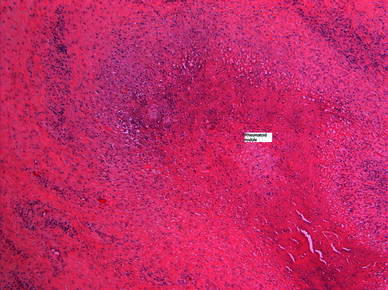
Hematoxylin and eosin ×100. Central fibrinoid necrosis rimmed by palisaded fibroblasts and histiocytes indicating a rheumatoid nodule

**Fig. 3:**
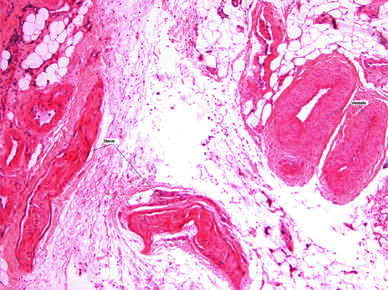
Hematoxylin and eosin ×25. Thickened blood vessels and nerve bundles that are rimmed by fibrous tissue consistent with Morton’s neuroma

Histology of the operative specimen revealed fibrinoid necrosis rimmed by palisaded histiocytes and fibroblasts, confirming the diagnosis of rheumatoid nodule (Fig. 2). It also showed thickened nerve bundles and blood vessels which were surrounded by fibrous tissue consistent with Morton’s neuroma (Fig. 3) in the left foot, and a rheumatoid nodule and unremarkable digital nerve bundles in the right foot.

She was subsequently tested for rheumatoid arthritis, and gave an equivocal result on RA assay and a strong positive result for anti-mutated citrullinated vimentin antibodies (anti-MCV) suggestive of seropositive rheumatoid arthritis. During follow-up, she developed symptoms of rheumatoid arthritis seven months after her first attendance at the foot and ankle clinic. She is currently in the care of the rheumatology team.

**Case 2.** A 37-year-old lady with a past medical history of rheumatoid arthritis presented to the foot and ankle clinic with pain and swelling in the second intermetatarsal space bilaterally. Clinical examination revealed splaying of the 2/3 toes bilaterally, with a positive Mulder’s click. Radiographs of the feet did not reveal any abnormality. A clinical diagnosis of Morton’s neuroma was made and the feet were operated on, one side at a time. Intraoperatively, large fluid-filled bursae were noted in the intermetatarsal spaces bilaterally, with nerves lying underneath and tethered to bursae.

Histology revealed Morton’s neuroma coexisting with rheumatoid nodules on both sides.

**Case 3.** A 38-year-old lady presented to the foot and ankle outpatient clinic with an 18-month history of pain over the plantar aspect of the third and fourth metatarsal heads. She was otherwise fit and healthy. Clinical examination revealed tenderness over the third and fourth MTP joints. A clinical diagnosis of synovitis of the third and fourth metatarsophalangeal joints was made, and an ultrasound scan was organized. Her blood work was normal, with an erythrocyte sedimentation rate (ESR) of 2, C-reactive protein (CRP) of 3, and a normal white cell count. The ultrasound scan revealed a hypoechoic mass in the third intermetatarsal space suggestive of a Morton’s neuroma. She underwent excision of the neuroma through a dorsal linear incision. Intraoperatively, a large neuroma was found with scar tissue. Histology revealed the presence of Morton’s neuroma and a rheumatoid nodule in the specimen. Her symptoms resolved following the surgery. She was tested positive on RA assay and was referred to rheumatologists for further management.

Between January 2004 and October 2008, 101 cases of Morton’s neuroma were operated on by two orthopedic surgeons with a special interest in foot and ankle surgery. Among these, histology revealed rheumatoid nodules in five cases. The chart below summarizes these cases. 

## Discussion

Morton’s neuroma can be a local manifestation of a generalized disease such as rheumatoid arthritis [5, 9]. The incidence of interdigital neuroma in rheumatoid arthritis patients was reported to be 1 in 520, with a female preponderance [9]. Rheumatoid synovitis and nodules producing symptoms mimicking Morton’s neuroma have been reported in the literature, but most of the cases were found in previously diagnosed rheumatoid arthritis patients [1, 9]. Awerbuch et al. [1] presented a series of 50 patients with Morton’s neuroma, and found that 24 % had rheumatoid arthritis at the time of diagnosis, 16 % developed rheumatoid arthritis over a follow-up period ranging from two months to 15 years, and an inflamed bursa in the intermetatarsal space was the first sign of rheumatoid arthritis in 8 %. In this series, out of 20 patients, a histological diagnosis of rheumatoid arthritis without any evidence of Morton’s neuroma was made for ten patients, of which seven were asymptomatic at presentation but three developed symptoms with four years of the initial presentation. Awerbuch et al. suggested seeking histological evidence of rheumatoid disease in all tissues excised in the surgical treatment of Morton’s neuroma, as they believed that rheumatoid disease is the basic etiology in a significant number of patients. In the senior author’s view, this is a rather interesting and unusual finding, and may be due to selection bias.

In a series by Vainio [9], performing prophylactic metatarsophalangeal synovectomy in rheumatoid arthritis patients resulted in a decrease in Morton’s neuroma operations. The distribution of the location of the neuroma in rheumatoid arthritis patients in this series was almost the same (in interspaces II–III and III–IV), in contrast to nonrheumatoid patients, in whom the III–IV interspace is more common. Vainio also suggested that metatarsalgia associated with rheumatoid arthritis should preferably be called Morton’s metatarsalgia, as the nerve changes in this group of patients are not typical of neuroma.

In our case series, out of three patients, one had a history of rheumatoid arthritis, and two patients were later diagnosed with seropositive rheumatoid arthritis after rheumatoid nodules were identified on a histological examination of the excised tissue. Our case series demonstrates that a rheumatoid nodule presents with symptoms similar to Morton’s neuroma. However, the number of cases in our series is too small to suggest that patients with II–III intermetatarsal neuralgia may be rheumatoid in origin, although the literature seems to suggest that view.

We therefore suggest that a nodule of rheumatoid origin should be considered in the differential diagnosis of Morton’s neuroma, not only in patients with a known history of rheumatoid arthritis but also in patients who present with symptoms of Morton’s neuroma and have atypical intraoperative findings. The presence of a rheumatoid nodule in the foot can be the first manifestation of rheumatoid arthritis, and requires prompt referral to the rheumatology team.
